# Barriers and Facilitators to PrEP Adherence among Transgender and Non-binary Individuals: A Mixed-Methods Analysis of Psychosocial Factors and Health Belief Model Constructs

**DOI:** 10.1007/s10461-025-04810-y

**Published:** 2025-07-11

**Authors:** D. Paltin, M. Prescott, J. Ma, S. Yeager, L. Ham, S. Serrano, J. Narez, J. Delgado, L. Burke, B. Gouaux, M. Beckwith, S. R. Morris, D. J. Moore, J. L. Montoya

**Affiliations:** 1 San Diego State University, University of California San Diego Joint Doctoral Program in Clinical Psychology, San Diego, CA USA; 2 University of California, San Diego, CA USA; 3 San Diego State University, San Diego, CA USA; 4 HIV Neurobehavioral Research Program, San Diego, CA USA

**Keywords:** PrEP adherence, HIV prevention, Transgender, Nonbinary, Motivational interviewing

## Abstract

Despite known benefits of Motivational Interviewing (MI) for medication adherence, its effectiveness in supporting pre-exposure prophylaxis (PrEP) adherence among transgender and nonbinary (TGNB) populations remains underexplored. This study applies mixed-methods analysis to understand PrEP adherence among TGNB individuals who received a daily individualized text-message intervention and phone-based MI for non-adherence to PrEP. Individuals who did not respond to three consecutive messages were identified as potential MI recipients. We had three objectives: (1) examine psychosocial differences between participants who needed MI (MI Indicated group; n = 81) versus those who did not (MI Not Indicated group; n = 48), (2) assess whether Health Belief Model (HBM) constructs were associated with PrEP adherence, and (3) identify adherence barriers and facilitators. This secondary analysis builds on primary intervention outcomes published in Morris et al. (J Acquire Immune Defic Syndr 91:453–459, 2022). We conducted multivariable linear regression on psychosocial measures and PrEP adherence, and inductive qualitative analysis on a subset of participants who completed at least one MI session (n = 60). Results were deductively mapped onto HBM constructs. Significant differences emerged between MI groups in HBM constructs, with the MI Indicated group reporting higher perceived HIV risk, stress, depressive symptoms, and poorer coping and self-efficacy. Findings highlight the need for personalized interventions to support PrEP adherence, mental health, and HIV risk perception among TGNB individuals. Future adherence interventions may benefit from assessing and addressing HBM constructs. To support the Ending the HIV Epidemic initiative, it is critical to enhance access to facilitators and mitigate barriers to PrEP adherence for this population.

## Resumen

A pesar de los beneficios conocidos de la Entrevista Motivacional (EM) para la adherencia a la medicación, su eficacia en la adherencia a la profilaxis preexposición (PrEP) entre poblaciones transgénero y no binarias (TGNB) sigue poco explorada. Este estudio aplica métodos mixtos para comprender la adherencia a la PrEP entre personas TGNB que recibieron una intervención diaria individualizada mediante mensajes de texto y EM telefónica por falta de adherencia. Quienes no respondieron a tres mensajes consecutivos fueron identificados como posibles destinatarias de EM. Tuvimos tres objetivos: (1) examinar diferencias psicosociales entre quienes necesitaron EM (grupo indicado; n = 81) y quienes no (grupo no indicado; n = 48), (2) evaluar si los constructos del Modelo de Creencias de Salud se asociaron con la adherencia, y (3) identificar barreras y facilitadores. Este análisis secundario se basa en resultados publicados en Morris et al. (J Acquire Immune Defic Syndr 91:453–459, 2022). Aplicamos regresión lineal multivariable sobre medidas psicosociales y adherencia, y un análisis cualitativo inductivo con quienes completaron al menos una sesión de EM (n = 60). Los resultados se mapearon según los constructos del Modelo de Creencias de Salud. Surgieron diferencias significativas entre los grupos: el grupo indicado reportó mayor riesgo percibido de VIH, estrés, síntomas depresivos y menor afrontamiento y autoeficacia. Los resultados subrayan la necesidad de intervenciones personalizadas que apoyen la adherencia, salud mental y percepción del riesgo de VIH. Futuras intervenciones podrían beneficiarse de abordar estos constructos. Mejorar el acceso a facilitadores y mitigar barreras a la PrEP es clave para apoyar la iniciativa ending the HIV Epidemic.

## Introduction

Pre-exposure prophylaxis (PrEP) is highly effective at preventing HIV transmission. When taken daily as prescribed, PrEP reduces the risk of HIV transmission through sex by about 99% and by 74% through injection drug use [1–3]. The standard dosing regimen is daily oral PrEP. However, event-driven dosing—taking PrEP before and after sex—is also an effective option for gay, bisexual, and other men who have sex with men (MSM) and transgender women, particularly for receptive anal intercourse. For individuals who inject drugs, daily dosing remains the recommended strategy. PrEP dosing recommendations can vary based on the mode of exposure, such as anal versus vaginal intercourse or injection drug use. Additionally, long-acting injectable PrEP is now available and may be especially beneficial for individuals facing barriers to daily adherence or consistent access to care. Despite the effectiveness of PrEP, many disproportionately affected communities with high HIV incidence have suboptimal PrEP awareness and limited uptake. Transgender (TG) and nonbinary persons (NB) are individuals whose gender identity does not align with the sex assigned to them at birth (transgender) or who do not identify exclusively as male or female (nonbinary). Transgender women are disproportionately impacted by HIV transmission, with worldwide HIV prevalence of 19.9% [4]. As of 2021, the odds of HIV infection is 66.0 more likely for transgender individuals when compared to all people over age 15 [4]. The high HIV prevalence among TGNB individuals is further compounded by their low uptake of PrEP [5–9]. One study from 2016 found that 62% of transgender women met the CDC’s PrEP needs threshold (i.e., individuals who do not have HIV, but may be exposed to HIV from sex or injection drug use), yet only 31% were aware of PrEP [10]. A more recent 2023 survey of TGNB individuals in a large Southern California hospital system found that while more than 75% of the participants were familiar with PrEP, less than 5% had taken PrEP [9]. The overall low PrEP uptake among TGNB may be understood through various individual-(e.g., lack of awareness about PrEP, concerns about side effects/drug interactions, perceived low risk)[11–14], interpersonal- (e.g., multiple forms of stigma related to sexual orientation, gender identity, drug use, and/or sexual behaviors, lack of social support, medical mistrust) [12, 14, 15], and structural-level factors (e.g., limited healthcare access, navigating healthcare experiences, and cost barriers) [12, 15–17] influencing access and decision-making regarding PrEP uptake. TGNB persons represent a population for which supporting PrEP uptake is paramount towards preventing HIV transmission [18], warranting more research to inform and scale-up implementation strategies and supportive interventions aimed at maximizing PrEP uptake and adherence tailored to TGNB persons [19, 20].

The research literature has elucidated a multitude of barriers and facilitators to PrEP adherence among TGNB. Behavioral science theories can assist research and practice by consolidating the multiple of factors influencing PrEP adherence. The Health Belief Model (HBM) [21, 22], for example, is a widely used model in health behavior interventions. As it relates to PrEP adherence, HBM posits that an individual’s level of adherence is a function of their perceived susceptibility to HIV (i.e., risk of acquiring HIV), perceived severity of HIV (i.e., negative consequences associated with an HIV diagnosis), perceived benefits (i.e., perceived effectiveness) of PrEP adherence to reduce susceptibility to HIV, perceived barriers to PrEP adherence, cues to action (i.e., stimulus needed to trigger the decision-making process to adhere to PrEP), and self-efficacy (i.e., confidence in one’s ability to adhere to PrEP). Previous research employing the HBM found that perceived vulnerability to HIV and belief in PrEP efficacy are strong motivators for PrEP adherence [21, 22]. Related to the concept of self-efficacy, a perceived sense of control over one’s health and responsibility for self-care has been shown to promote PrEP adherence among transgender women [23].

Motivational interviewing (MI), a therapeutic technique originally developed in the context of addiction treatment, may be a promising strategy to promote PrEP uptake and adherence for TGNB individuals. MI involves patient-centered communication skills to address negative health behaviors and support positive health behavior changes by targeting ambivalence about change [24]. Considered a collaborative method of communication and information exchange, MI is designed to support intrinsic motivation for and commitment to behavior change and has received empirical support on its utility to reduce or eliminate health-risk behaviors across diverse settings and populations [25–27]. Abundant empirical evidence shows that MI interventions are efficacious for people with substance use disorders and improving clinical outcomes and health behaviors, such as weight loss, lowering blood pressure and cholesterol, and promoting medication adherence [28]. Specific to medication adherence, incorporating MI skills has been an effective tactic to increase medication adherence among people with HIV [29]. The potential for MI to promote health behavior change is particularly noteworthy for people exposed to HIV. Despite the usefulness of MI for medication adherence, the potential usefulness of MI to support PrEP adherence among TGNB has been understudied.

To address this gap, we conducted a mixed-methods study among a diverse sample of TGNB folks who could benefit from HIV prevention options in Southern California. Our study features a secondary analysis of primary intervention outcomes reported by Morris et al. (2022) [30], which revealed no statistically significant differences in PrEP adherence between individuals who received an individualized text messaging intervention alone versus those who received the individualized text message intervention plus phone-based MI when indicated (i.e., MI was indicated after three consecutive days of a participant being unresponsive to the text messaging intervention). The current study sought to explore why the addition of MI might not have achieved the expected positive effect on PrEP adherence. Thus, our analysis had three primary aims: (1) to examine differences in psychosocial variables between individuals with indications for MI and those without, (2) to assess the extent to which the HBM framework could account for variance in PrEP adherence, and (3) to identify barriers and facilitators to PrEP adherence and intervention engagement. Our hypotheses posited that individuals who needed MI would exhibit greater psychosocial needs compared to their counterparts who did not need MI. Furthermore, we anticipated that constructs of the HBM would be associated with PrEP adherence. Our approach offers a comprehensive understanding of PrEP adherence for an understudied population, providing rich insights into individual experiences, perceptions, and contextual factors that future clinicians and researchers may build on.

## Methods

### Study Sample and Recruitment

The present study is a secondary data analysis of quantitative and qualitative data from 255 participants who participated in the iM-PrEPT (individualized Texting for Adherence Building (iTAB) Plus Motivational Interviewing for PrEP Adherence in Transgender Individuals) study [30]. iTAB is a behavioral intervention that uses human-generated personalized text messages. iTAB intervention for PrEP adherence is recognized by the CDC compendium of evidence-based interventions and best practices for HIV prevention. iM-PrEPT combines iTAB with motivation interviewing for transgender individuals. Specifically, the present analysis focuses exclusively on iM-PrEPT participants who were randomized to iTAB + MI (i.e., the active arm of the intervention). This secondary analysis is limited to participants in the active intervention arm in order to examine variability in adherence outcomes based on whether participants were indicated for motivational interviewing (MI) as part of the adaptive intervention. iM-PrEPT participants were recruited from five sites across Southern California between June 2017 and September 2020. Recruitment was conducted using a variety of methods that engaged the TGNB community and is described in detail in the primary analysis article by Morris et al. (2022) [30]. These included collaboration with a Trans Community Advisory Board, hosting trans-centered events, outreach through a study website and social media, tabling at community gatherings, partnerships with local organizations and providers, and word-of-mouth referrals. Individuals were eligible to participate if they were (a) HIV-negative, (b) at elevated risk for contracting HIV, (c) transgender or nonbinary, (d) 18 years of age or older, and (e) spoke English or Spanish. Participants’ HIV-negative status was confirmed by a fourth-generation antigen–antibody assay or a third-generation antibody assay plus an HIV nucleic acid amplification test (NAAT). Individuals were considered at elevated risk for HIV if they endorsed one or more of the following at screening: (1) having at least one partner with HIV in a sexual relationship lasting at least 4 weeks in the past year; (2) anticipated condomless anal or vaginal sex with an assigned-male-at-birth (AMAB) partner in the next 3 months; (3) any AMAB partners in the past 12 months and at least one of the following: (a) any condomless anal or vaginal sex in the past 12 months; (b) any sexually transmitted infection (STI) in the past 12 months; (c) exchange of money, gifts, shelter, or drugs for sex; and/or (d) HIV post-exposure prophylaxis use in the past 12 months. Participants provided written informed consent to participate in this University of California, San Diego IRB-approved study and had follow up visits every 12 weeks up to 48 weeks.

### Intervention and Data Collection

All participants received study-provided PrEP (oral TDF/FTC 200/300 mg daily) along with the Individualized Texting for Adherence Building (iTAB) intervention to support and monitor PrEP adherence. Through the iTAB system, participants received personalized daily health promotion and “factoid” messages at an individually selected time to promote PrEP adherence. iTAB is a behavioral intervention that uses personalized text messages; the messages are typically tailored to the individual’s needs, but the intervention itself is not powered by AI. Instead, it relies on human input for creating the messages using pre-programmed templates and guidelines. These messages are followed by the question: “did you take your [dose] today?” to which participants could respond with the following text options: (Y) yes, (N) no, or (P) postpone. If a participant opted to postpone, they would receive a follow up message one hour later. After responding to the text reminder, participants received a variable message reinforcing their medication adherence (e.g., “Good job!”) or a message encouraging adherence (e.g., “Please take your PrEP ASAP”). Either a “No” or non-response on a given day was considered a missed dose of PrEP for that day. We adopted this approach based on existing iTAB literature, which suggests a significant positive relationship between responsiveness to text messages and biological measures of PrEP adherence.

Participants were randomized 1:1 to receive iTAB intervention with or without brief MI for suboptimal adherence for 48 weeks. Participants were considered to have suboptimal PrEP adherence if they had three consecutive days of responding “no” and/or not responding to the iTAB text reminders. Participants who did not respond to repeated MI phone calls, or indicated that they would be unavailable, were switched to a monthly call cycle until their agreed return date or until they re-engaged by responding to iTAB messages. Brief MI was delivered by a research coordinator from UC San Diego who had received 16 h of targeted MI training from a psychologist specialized in MI. The research coordinator who conducted the MI phone call varied depending on staff availability and turnover over the course of the study. During MI sessions, research coordinators used MI core components to discuss barriers and facilitators of PrEP adherence, and adherence enhancement techniques with participants. Phone calls, including their duration, were not recorded. Research coordinators completed templated forms summarizing the content of MI sessions, which followed a scripted format. These summary forms included all script points. Conversations were also coded as they occurred. If a participant acknowledged any barriers to taking PrEP, the research coordinator was instructed to code the barrier and participants’ comments in one of the six pre-established categories: phone/text issues; iTAB issues; issues remembering to take PrEP; having competing priorities; issues with having and keeping medication nearby; being unmotivated to take medication; or other. These barrier codes were predetermined based on previous iTAB literature. If “Other” was selected, then the research coordinator was instructed to describe the barrier in a text field. As a part of the MI interview, research coordinators also recorded the “strengths” (i.e., positive attributes or skills that promote intervention engagement and PrEP adherence) and “solutions” (i.e., ways in which the participant troubleshoots, or problem solves their barriers to PrEP adherence). The strengths and solutions fields of the phone call summary were open-ended, allowing the research coordinator to capture a wide range of insights. The responses captured in the “Other” field, as well as the “Strengths” and “Solutions” field, were the focus of the current qualitative analysis.

In addition to receiving the daily iTAB intervention, participants attended in-person screening and baseline visits, and a telephone check-in at week 2. Participants completed a computer-assisted self-interview (CASI) at baseline at the same time as the demographic and psychosocial measures represented in the HBM. Participants completed in-person HIV testing at weeks 12, 24, 36, and 48, in accordance with CDC recommendation that people taking oral PrEP get tested for HIV every three months [31]. PrEP adherence was measured at weeks 12 and 48 using dried blood spot testing to confirm Tenofovir-Diphosphate (TFV-DP) levels. Participants were classified as having ‘perfect adherence’ if their TFV-DP concentrations exceeded 1246 (fmol/punch) [32, 33]. Concentrations between 1246 and 719 (fmol/punch) were categorized as ‘adequate adherence,’ while those below 719 (fmol/punch) were categorized as having ‘suboptimal adherence.’

### Measures

#### Demographic Characteristics

Self-reported demographic data were collected on age, gender, sexual orientation, race/ethnicity, employment status, education level, and relationship status. Age was recorded as a continuous variable. Gender was self-reported as transgender women, transgender men, or gender diverse/nonbinary. Hormone therapy use was recorded as a binary variable (yes/no). Sexual orientation was classified as LGBTQ + or heterosexual, with counts and percentages for each. Race/ethnicity categories included Latinx/Hispanic, non-Latinx/Hispanic White (labeled “White”), multiracial or other, non-Latinx/Hispanic Asian (labeled “Asian”), and non-Latinx/Hispanic Black/African American (labeled “Black/African American”). Employment status was categorized as employed, unemployed, or other (including retired, unable to work, or student). Education level was grouped into three categories: high school diploma/GED or less, some college/technical training, and bachelor’s degree or higher. Relationship status was classified as single, in a monogamous relationship, or in an open relationship.

**Fig. 1:**
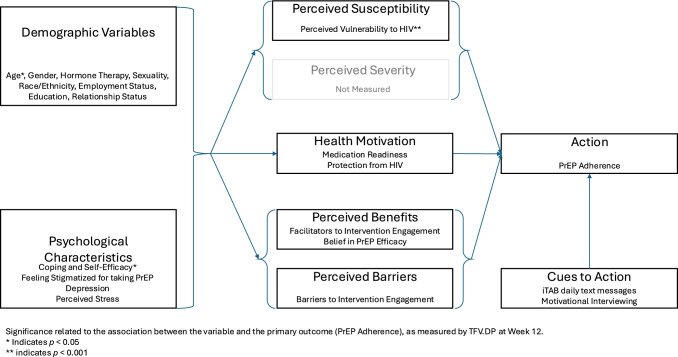
Significance related to the association between the variable and the primary outcome (PrEP Adherence), as measured by TFV.DP at Week 12. * Indicates *p* < 0.05. ** indicates *p* < 0.001

We used the HBM [21, 22] as a guide to narrow our focus to the theoretically most salient influences on PrEP adherence (see Fig. 1).

#### Perceived Susceptibility to HIV

Participants rated their likelihood of acquiring HIV in the next few months under three different conditions: (i) without any prevention strategies, (ii) with current prevention strategies, and (iii) with daily PrEP. Participants rated their likelihood of acquiring HIV for each of these conditions on a scale from 0 “no chance/won’t happen” to 100 “definitely will happen.”

#### Perceived Benefits of PrEP Adherence

To assess perceived PrEP effectiveness, participants were asked “On a scale of 0 to 100, how effective do you believe PrEP to be in preventing HIV infection?” Participants provided a response on a scale from 0 (“PrEP will not prevent HIV infection”) to 100 (“PrEP will always prevent infection”), with higher scores indicating a greater perception of PrEP effectiveness.

#### Health Motivation

The Medication Readiness Scale was used to measure psychological readiness for initiating and adhering to PrEP medication [34]. This scale was adapted for PrEP medication, in which the wording was slightly modified as “PrEP pills’’ replaced the items and instructions that said, “HIV pills.” To measure the construct of self-rated protection from HIV while taking PrEP, participants were asked, “Would you feel protected from getting HIV?” and could respond “Yes definitely,” “Yes probably,” “No probably not,” and “No definitely not.” The 10-item scale was scored by the sum of all items, with higher scores indicating greater readiness for engaging with PrEP medication.

#### Psychological Assessment

The Patient Health Questionnaire-9 (PHQ-9) was used as a self-report measure of depressive symptom severity [35]. The PHQ-9 asked participants to rate how often they have been bothered by nine depressive symptoms over the previous two-week period, which were summed together for a total sum score ranging from 0– 27, with higher scores indicating higher depressive severity.

The Coping and Self-Efficacy Scale (CSES) measured participants’ coping behaviors in the face of stressors [36], which includes 26 coping behaviors, such as “take your mind off unpleasant thoughts.’’ The CSES total score ranged from 0 to 260, with higher scores indicating higher utilization of coping behaviors when faced with stressors.

The Perceived Stress Scale [37] assessed appraisal of stress in the previous month. The scale consists of ten items that were summed together, with a total score ranging from 0 to 40. Higher scores indicated higher perceived stress during the previous month.

Feeling stigmatized for taking PrEP was assessed using a single item that asked participants, “Would you feel stigmatized for taking PrEP?” with the following answer choices: “Yes definitely,” “Yes probably,” “No probably not,” and “No definitely not.”

### Cues to Action

#### Primary Predictor: Motivational Interviewing Indication

Participants within the iTAB + MI for suboptimal adherence arm were indicated for an MI session during the intervention period after three consecutive days of responding “no” and/or not responding to the iTAB system. In the present analysis, participants were dichotomized as MI Indicated if they received any MI indications over the study period, and MI Not Indicated only if they received none.

#### iTAB Daily Text Messages

iTAB texting behaviors during the intervention period encompassed three metrics: (a) the total count of MI indications, (b) the total number of affirmative “Y” responses to iTAB text messages, and (c) the total number of any valid responses to the iTAB text messages, which could include “Y,” “N,” or “P.” iTAB-reported adherence was calculated for each participant as the proportion of “Y” text responses submitted between the following periods: weeks 0–12, weeks 13–48, and weeks 0–48.

### Study Outcomes

#### Primary Outcome: PrEP Adherence

Dried blood-spot testing was conducted at weeks 12 and 48 to measure levels of TFV-DP to approximate PrEP adherence and compare adherence across time. Participants were considered to have “perfect adherence” if their TFV-DP concentrations were greater than 1246 (fmol/punch) [32, 33]. Those with concentrations between 1246 and 719 (fmol/punch) were considered to have “adequate adherence” and those less than 719 (fmol/punch) were considered to have “suboptimal adherence.” As PrEP Adherence was the primary outcome of interest, participants missing dried blood spot data at both weeks 12 and 48 were excluded from analyses.

#### Secondary Outcome: Study Retention

Study retention was defined as the proportion of participants still enrolled at weeks 12 and 48 and was calculated for each of the dichotomized MI groups. Participants were considered enrolled if they completed dried blood spot testing at weeks 12 and 48.

### Analytic Approach

#### Quantitative Analysis

We examined group differences in PrEP adherence and intervention engagement between MI Indicated vs. MI Not Indicated groups using parametric tests for normally distributed variables (t-tests for continuous variables and chi-squared tests for categorical data) and non-parametric tests for non-normally distributed variables (Kruskal–Wallis tests, Wilcoxon Rank Sum tests, and Spearman’s rank correlations). Demographic variables and variables of the HBM were considered as potential covariates in stepwise linear regressions by examining their bivariate associations with PrEP adherence (i.e., TFV levels), the primary outcome variable. Stepwise linear regression was used to identify key predictors associated with the outcome variable while minimizing model complexity and controlling for confounders. This approach allowed for the systematic inclusion and exclusion of variables based on their statistical significance, ensuring that only the most relevant predictors were retained.

A multivariable linear regression model examined the relationship between baseline psychosocial measures and PrEP adherence at week 12. Variables demonstrating an association with PrEP adherence at *p* < 0.2 in bivariate analyses were included in multivariable regression models [38]. A significance level of p < 0.2 was chosen for variable inclusion in the multivariable regression models to balance the identification of potentially important predictors while avoiding the exclusion of variables with moderate effects. This more liberal threshold is commonly used in exploratory analyses to capture variables that may have practical significance, even if their associations do not meet stricter significance levels (e.g., p < 0.05).

After including all relevant covariates, we performed backwards elimination to identify the best fitting and most parsimonious regression model of PrEP adherence. Lastly, we entered MI Group (Indicated vs Not Indicated) into the model as an a priori primary predictor of interest. All quantitative analyses were completed using JMP Pro 17 with two-tailed tests and a pre-determined alpha of 0.05.

#### Qualitative Analysis

The qualitative analysis focused exclusively on a subset of participants in the MI Indicated group who completed at least one MI session (n = 51). The MI call logs were analyzed to identify barriers (n = 139 entries) and facilitators (n = 99 entries) related to PrEP adherence and/or iTAB engagement. Analysis of barriers and facilitators initially followed an inductive approach, and then deductively matched to the HBM constructs of perceived barriers and perceived benefits, respectively.

The MI call logs were exported to Microsoft Excel and analyzed through inductive content analysis [39]. Open-ended entries in the “other” category of the MI call log were examined for barriers, while “strengths” and “solutions” were examined for facilitators.

Two clinical psychology doctoral students with advanced training in qualitative methods independently reviewed excerpts and developed potential codes. Upon achieving full consensus in code definitions and observing no additional emerging codes to ensure data saturation, the two coders applied the reconciled list of codes to the excerpts independently, held consensus meetings, and addressed all discrepancies in code application. 100% inter-rater reliability was achieved between the two coders (authors DP and JM). Coders included an Asian cisgender male and a White cisgender female researcher who both hold sexual minority identities. This positionality statement reflects the authors’ awareness that coders’ social identities may influence the analysis and interpretation of qualitative results.

## Results

### Quantitative Results

#### Demographic Characteristics

Overall, participants (N = 129) were predominantly in their mid-20’s to mid-30’s (median age = 29.0, interquartile range [IQR] = 24.0– 37.0) and consisted of transgender women (58.6%), transgender men (23.4%), and gender diverse/non-binary individuals (18.0%). More than half of the sample identified as LGBTQ (60.2%) and reported being single (62.5%). Participants were diverse in terms of race/ethnicity (72% racial/ethnic minority), employment status, and education level (see Table 1). Of the baseline demographic characteristics, the MI Indication groups (MI Indicated vs MI Not Indicated) only differed by race/ethnicity, such that the MI Not Indicated group had a higher proportion of Latinx/Hispanic individuals (56% vs 44%, *p* = 0.04).

**Table 1:** Baseline demographic characteristics of participants by motivational interviewing (MI) indication group

	MI Indicated N = 81 (62.8%)	MI Not Indicated N = 48 (37.2%)	*p*-Value
Age, median [IQR]	28.5 [24.0–36.5]	30 [24.0–37.0]	0.61
Gender, n (%)			0.70
Transgender woman	45 (60.0)	30 (40.0)	
Transgender man	19 (63.3)	11 (36.7)	
Gender Diverse/Nonbinary	16 (69.6)	7 (30.4)	
On Hormone Therapy, n (%)	52 (61.9)	32 (38.1)	0.78
Sexual Orientation, n (%)			0.28
LGBQ +	51 (66.2)	26 (33.8)	
Heterosexual	29 (56.9)	22 (43.1)	
Race/Ethnicity, n (%)			
Latinx/Hispanic	19 (44.2)	24 (55.8)	0.04
White	24 (66.7)	12 (33.3)	
Multiracial or Other	15 (79.0)	4 (21.1)	
Asian	12 (75.0)	4 (25.0)	
Black/African American	10 (71.4)	4 (28.7)	
Employment Status, n (%)			0.07
Employed	33 (64.7)	18 (35.3)	
Unemployed	26 (52.0)	24 (48.0)	
Other (e.g., retired, unable to work, student)	21 (77.8)	6 (22.2)	
Education Level, n (%)			0.25
Highschool Diploma, GED, or less	22 (52.4)	20 (47.6)	
Some College or Technical Training	39 (68.4)	18 (31.6)	
Bachelor’s Degree, Advanced Degrees	19 (65.5)	10 (34.5)	
Relationship Status, n (%)			0.67
Single	48 (60.0)	32 (40.0)	
In Monogamous Relationship	16 (69.6)	7 (30.4)	
In Open Relationship	15 (65.2)	8 (34.8)	

Row percentages are used to highlight how characteristics differ across study conditions, which aligns with the comparative focus of our analysis

*LGBTQ* Lesbian, Gay, Bisexual, Transgender, Queer; *IQR* interquartile range; *GED* General education development

During the 48-week study, 62.8% of the sample (n = 81) was classified into the MI Indicated group. Participants in the “MI Completed” group participated, on average, in two MI sessions (SD = 1). The remaining participants were classified into the “MI Not Indicated” group (n = 48). Within the MI Indicated group, the mean number of MI indications at week 12 was 3.41 (SD = 3.28). The percent of affirmative “Y” responses, indicating PrEP adherence via text message, among the MI Not Indicated group was significantly greater than in the MI Indicated group (median = 95.8% vs 74.4%; z = 4.55, *p* < 0.01). The MI Not Indicated group also had a higher number of valid responses to the iTAB text messages compared to the MI Indicated group (mean = 69.2 vs 56.8; z = 4.44, *p* < 0.01), see Table 3.

**Table 3:** Baseline PrEP preferences and psychosocial measures by motivational interviewing (MI) indication group

Health belief model	MI Indicated (N = 81)	MI Not Indicated (N = 48)	*p*-Value
Perceived susceptibility			
Perceived vulnerability to HIV (%), median [IQR]			
Self-rated probability of HIV infection without any prevention strategies^a^	50.0 [24.3–70.0]	50.0 [16.5–80.0]	0.95
Self-rated probability of HIV infection with current prevention strategies^b^	25.0 [8.5–50.0]	10.0 [1.0–28.0]	0.03
Self-rated probability of HIV infection with daily PrEP^c^	15.0 [4.0–42.3]	6.5 [0.8–38.8]	0.15
Perceived Benefits			
Self-rated PrEP efficacy^†^, median [IQR]^d^	91 [85–97]	90 [75–97]	0.22
Health Motivation			
Medication Readiness Scale, median [IQR]^e^	31.0 [26.5–38.5]	35.5 [30.0–38.8]	0.05
Feels protected from HIV while taking PrEP, n (%)^f^	72 (61.0)	46 (39.0)	0.08
Psychological characteristics			
Feels stigmatized for taking PrEP, n (%)^g^	11 (61.1)	7 (38.9)	0.79
PHQ-9, median [IQR]^h^	9.0 [4.0–14.5]	6.0 [1.0–10.0]	0.02
Perceived Stress Scale, mean (SD)^i^	28.5 (6.7)	24.8 (7.3)	< 0.01
Coping and Self-Efficacy Scale, mean (SD)^j^	81.5 (28.4)	92.3 (22.2)	0.03

^†^*on a scale from 0–100%, with 100% indicating PrEP will always prevent HIV infection*

PHQ-9 = Patient Health Questionnaire, 9 item

^a^N = 106; ^b^N = 107; ^c^N = 108; ^d^N = 115; ^e^N = 125; ^f^N = 124; ^g^N = 120; ^h^N = 105; ^i^N = 123; ^j^N = 124

#### Psychosocial Measures by MI Indication Group

Analyses revealed significant differences between the MI indication groups for the HBM constructs of perceived susceptibility and psychological characteristics at baseline. Specifically, the MI Indicated group estimated a significantly greater likelihood of acquiring HIV with their current prevention strategies compared to the MI Not Indicated group (z = − 2.19, median = 25.0% vs 10.0%, *p* = 0.03). The MI Indicated group reported higher stress (mean = 28.5 vs 24.8), depressive symptoms (median = 9.0 vs 6.0), and poorer coping and self-efficacy (mean = 81.5 vs 92.3), compared to the MI Not Indicated group, respectively (all *p-*values < 0.05). The MI Indicated group was not statistically different from the MI Not Indicated group on other PrEP preferences and psychosocial characteristics assessed at baseline (e.g., belief in PrEP efficacy, medication readiness, feeling protected from HIV or stigmatized for taking PrEP); see Table 2.

**Table 2:** Study outcomes by motivational interviewing (MI) indication

Outcome	MI indicated (N = 81)	MI not indicated (N = 48)	Test statistic	*p*-Value
Study retention, n (%)				
Retention at Week 12 ^a^	57 (70.4%)	31 (65.4%)	χ^2^ = 0.47	0.50
Retention at Week 48 ^b^	24 (29.6%)	18 (37.5%)	χ^2^ = 0.84	0.36
iTAB-Reported Adherence, median % [IQR]				
Text-reported PrEP adherence (week 0 to 12) ^c^	74.4 [53.7–87.8]	95.8 [82.1–98.8]	z = 4.53	< 0.01
Text-reported PrEP adherence (week 13–48) ^d^	64.3 [30.4–83.7]	92.7 [51.6–96.0]	z = 1.25	0.21
Text-reported PrEP adherence (week 0 to 48) ^d^	70.2 [40.0–84.9]	93.6 [24.4–96.7]	z = 1.47	0.14
PrEP Adherence, median [IQR]				
TFV at Week 12	1330.4 [720.5–2010.6]	1883.2 [1175.7–2382.5]	z = 1.86	0.06
TFV at Week 48	1423.5 [296.0–1954.6]	1778.8 [1143.3–2518.5]	z = 1.26	0.20

Text-reported PrEP adherence was measured by the proportion of “Y” responses to iTAB text messages regarding PrEP adherence

*TFV* Tenofovir diphosphate

^a^N = 88, ^b^N = 42, ^c^N = 121; ^d^N = 64

#### PrEP Adherence by MI Indication Group at Weeks 12 and 48

At week 12, 68.8% of the total sample had biologic PrEP adherence data available (70.4% of the MI Indicated group and 65.4% of the MI Not Indicated Group). At week 48, only 32.5% of the total sample had biologic PrEP adherence data (29.6% of the MI Indicated group and 37.5% of the MI Not Indicated Group). Our study experienced high retention during the first twelve weeks, followed by a decline as the study progressed. By 48 weeks, attrition rates were consistent with those typically observed in HIV prevention studies, with approximately 50% of participants remaining. Median (IQR) TFV values at week 12 were 1330.4 (720.5– 2010.6) for the MI Indicated group and 1883.2 (1175.7– 2382.5) for the MI Not Indicated group (see Table 3). TFV values greater than 1246 correspond to excellent PrEP adherence (i.e., 5 or more doses per week). Differences in PrEP adherence by MI Indication group approached significance at week 12 (z = 1.86, *p* = 0.06), such that the MI Indicated group had lower TFV levels than the MI Not Indicated group. There was no statistically significant difference in PrEP adherence between MI Indication groups at week 48 (z = 1.23, *p* = 0.20).

#### Association of Demographic Variables and HBM Constructs with PrEP Adherence at Week 12

Due to significant attrition by week 48, PrEP adherence at week 12 was the primary outcome for multivariable models. Of the various demographic and HBM variables measured in this study, only age (Spearman’s rho = 0.25,* p* = 0.02), coping and self-efficacy (rho = 0.22, *p* = 0.04), and perceived vulnerability to HIV (rho = − 0.39, *p* < 0.01) were significantly correlated with PrEP adherence at week 12 (see Fig. 1). These variables were therefore considered for inclusion as predictors in the multivariable model of PrEP adherence in addition to MI indication group.

The final multivariable model predicting PrEP adherence at week 12 included age, perceived vulnerability to HIV, and MI Indicated group (see Table 4). The overall model fit was statistically significant (F(3, 77) = 6.67, R^2^ = 0.21, *p* < 0.001). Higher perceived vulnerability to HIV (β = − 11.50, *p* = 0.003) was significantly negatively associated with PrEP adherence, meaning as perceived vulnerability to HIV increased, PrEP adherence decreased. Age (β = 15.25, *p* = 0.097) and MI Indicated group (β = 331.7, *p* = 0.092) did not have significant effects on PrEP Adherence.

**Table 4:** Multivariable Model Predicting Week 12 PrEP Adherence

Variable	β	SE	*t*	*p*-value	95% CI
Age (years)	15.25	9.07	1.68	0.097	[− 2.83–33.33]
Perceived Vulnerability to HIV (%)	− 11.50	3.69	− 3.12	0.003	[− 18.83– − 4.16]
MI Not Indicated Group (ref. MI Indicated)	331.70	194.27	1.71	0.092	[− 55.38–718.79]

Overall model fit, (F(3, 77) = 6.67, R2 = 0.21, *p* < 0.001)

*CI* confidence interval

#### iTAB Texting Behaviors and PrEP Adherence

Across MI Indication groups at week 12, none of the iTAB texting behavior metrics were associated with PrEP adherence (*p*’s > 0.05). Across MI Indication groups at week 48, two texting behavior metrics were significantly associated with PrEP adherence. Specifically, the total count of “Y” responses indicating PrEP adherence via text message (Spearman’s rho = 0.56) and the total count of any valid response (rho = 0.52) were correlated with PrEP adherence at week 48 (*p*’s < 0.05). In follow-up analyses, associations between texting behaviors and PrEP adherence were examined separately within the MI Indicated and MI Not Indicated groups. In the MI Indicated group, both the total number of “Y” responses indicating PrEP adherence via text message and total valid responses were positively correlated with PrEP adherence at week 48 (rho = 0.61 and rho = 0.56, *p’*s < 0.05, respectively). No statistically significant associations were observed between texting responses and PrEP adherence in the MI Not Indicated group (*p*’s > 0.05).

### Qualitative Results

#### *Barriers to PrEP Adherence and iTAB Engagement (n* = *139 entries)*

The most commonly reported challenge was *competing priorities* (n = 64), in which participants either forgot to reply to iTAB text messages or faced competing priorities that interfered with responding to texts. *Medically related barriers* (n = 23) was the second most endorsed barrier, identified as a concern by participants who faced medical impediments, including PrEP side effects, unrelated health issues, wait times for test results, recent surgeries, hospitalization, inpatient care, insurance limitations, and logistical difficulties attending appointments. *Text message burden* (n = 19) emerged as the third most noteworthy barrier for a subset of participants who grappled with factors such as receiving too many texts, disliking texting, finding messages annoying, experiencing long intervals between texts, or having their messages turned off. On average, participants were sent 84 text message prompts over the course of 12 weeks and 333 prompts over the course of 48 weeks.

Moreover, participants shared *study-related issues* (n = 16) as a challenge to PrEP adherence, especially among those who encountered problems with iTAB, technology issues, IT-related problems, not receiving texts, usability issues, misunderstanding how to engage in the intervention, not comprehending texting expectations or language-related difficulties.

*Changes in routine* (n = 15) was a fifth factor affecting text communication and medication adherence. Instances of traveling without phones or medication, and modifications to daily schedules, work commitments, or routines contributed to suboptimal participation in the intervention and PrEP adherence. *No phone* (n = 14) was reported by participants who experienced issues related to losing their phone or phone service, phone breakage, or phone restrictions at work. *Emotional and social distress* (n = 12) indicated that participants reported interpersonal dilemmas and psychological or mental health challenges that hindered text message responses or adherence to daily PrEP medication.

A smaller subset of participants *chose to stop PrEP* (n = 8) due to a perceived lower risk of HIV from abstaining from sex or having a monogamous partner, resulting in reduced PrEP usage or intervention engagement. Finally, five participants reported not having PrEP medication (i.e., *no meds)* due to lapses in refills, running out of medication, misplacement, giving it away, or leaving it somewhere inaccessible. See Table 5 for full code definitions and representative excerpts.

**Table 5:** Barriers and facilitators to intervention engagement from motivational interviewing (MI) call logs

Perceived barriers (*N* = 139 Excerpts)		
Type of Barrier (N)	Definition	Sample Excerpt from MI Call Log
Competing Priorities (N = 64)	Participant reported that they either forgot to reply to the iTAB text messages or that they had competing priorities or demands off their time that interfered with them replying to texts	“ignored texts…due to competing priorities”
Medically Related Barriers (N = 23)	Participant reported a health concern like side effects from prep, health issues unrelated to prep, waiting for test results, or recent surgery. Participant was unable to participate in intervention due to hospitalization, inpatient treatment, job loss, accessibility issues making or keeping appointments	“hospitalized for 10 days and unable to respond to texts”
Text Message Burden (N = 19)	Participant had trouble responding to text messages because the receive too many texts per day, they don’t like texting in general, they find the text messages annoying, text messages are too far apart, or they turned messages off	“they get so many texts a day that they sometimes forget or can’t reply until the next day”
Study-related issues (N = 16)	Problems with iTAB, tech issues, IT issues, not receiving texts, usability of iTAB. Participant did not understand how to engage correctly in the intervention, did not understand texting expectations or how to reply, the intervention was not in the correct language	“She did not understand that you are supposed to respond to texts every day”
Changes in Routine (N = 15)	Participant is traveling out of town without phone, couldn’t receive or reply to texts, and/or did not bring their study meds. participant reported a change in their regular schedule, work schedule, or routine that prevented them from participating in the intervention or taking prep as regularly as planned	“traveling and wasn’t responding to any text messages.”
No Phone (N = 14)	Participant lost their phone or phone service, or their phone broke. Also, phone not allowed at work	“forgot phone a few days and forgot to respond to messages.”
Emotional and Social Distress (N = 12)	Participant reports interpersonal challenges, with partner or family, that interfere with their participation in the intervention. participant reported psychological or mental health issues that interfered with their participation in the intervention (e.g., poor mood, grief, depression, feeling uncomfortable, nightmares, feeling overwhelmed, developing negative associations with PrEP)	“not responding to MI due to mental health”
Choosing to stop PrEP (N = 8)	Participant reports lower risk of HIV that results in either reduced prep or reduced intervention engagement (e.g., not having sex or having a monogamous partner). Participant self-elected to stop taking prep or take a break from prep	“They decided to stop taking PrEP”
No Meds (N = 5)	Participant does not have meds due to lapse in refill/ran out, losing medication, giving it away, or leaving it somewhere inaccessible for a period	“lost study medication for 1.5 weeks.”
Perceived benefits / facilitators (*N* = 99 Excerpts)		
Code N	Definition	Sample Excerpt
Found Text Messages Helpful (N = 54)	Participants found text messages to be helpful reminders, they specifically mentioned features related to customization of text timing and alternative response options	“This study is helping her regularly take her PrEP more regularly than ever before. She finds the text messages really useful”
Perceived Benefits of PrEP (N = 37)	Participant reports psychological benefits of taking PrEP daily (e.g., peace of mind, feeling safe), taking prep because of heightened-risk evaluation, describes PrEP importance	“She feels safer using PrEP”
Additional Strategies (N = 33)	Participants reported using alarms, pill box, other tools, keeping meds on them or with them, or using social support for accountability to take their meds	“He fills pill boxes for the month, and then takes that week’s pill box with him”
Routines (N = 22)	participant described that taking prep is an express part of their daily routine (e.g., morning, evening, with a meal, before bed) and this helps them remember to take it every day	“She takes [PrEP] before bed and taking it has become part of her routine.”
Taking PrEP with Other Medications, Including Hormones (N = 10)	Participant described taking PrEP with other daily medication or with hormones	“Taking PrEP everyday with their HRT”

#### *Facilitators to PrEP Adherence and iTAB Engagement (n* = *99 excerpts)*

The most frequently reported facilitator was the perceived helpfulness of text messages (*found text messages helpful* [n = 54]), with participants finding them to be valuable reminders. Participants appreciated features like customizable text timing and alternative response options, contributing to a positive experience with the intervention. Thirty-seven participants reported psychological benefits associated with daily PrEP usage (*perceived benefits of PrEP*), such as peace of mind and feeling safe. Additionally, some participants cited a heightened-risk evaluation as a motivator for taking PrEP (e.g., engagement in condomless sex with multiple partners), underscoring the perceived importance of the intervention.

*Additional adherence strategies* (n = 33) were adopted by many participants, including the use of alarms, pillboxes, tools, keeping medications on hand, and leveraging social support for accountability. These additional strategies, many of which could be conceptualized as “cues to action,” played a crucial role in ensuring consistent adherence to PrEP. Furthermore, *establishing routines* (n = 22) emerged as a facilitator, as noted by participants who integrated taking PrEP into their daily schedules, whether it be in the morning, evening, with a meal, or before bed. This routine-oriented approach contributed to sustained daily adherence. A small portion of participants highlighted the facilitative aspect of *taking PrEP with other medications, including hormones* (n = 10). This concurrent medication management strategy was found to be effective for a subset of participants and is particularly salient for this sample of transgender and non-binary individuals. See Table 5 for full code definitions and representative excerpts.

## Discussion

This mixed-method study explored factors associated with PrEP adherence among participants randomized to receive the iTAB intervention with brief MI for suboptimal adherence in the iM-PrEPT study [30]. Results from this mixed-method study highlighted that TGNB with suboptimal PrEP adherence who need additional MI support were associated with greater perceived HIV susceptibility, greater depressive symptoms and stress, and poorer coping/self-efficacy. This study fills research gaps on PrEP adherence support for TGNB individuals potentially exposed to HIV. Using a mixed-methods approach, we identified characteristics of individuals who may benefit from text message reminders and MI, highlighting the need for tailored mental health strategies to address PrEP adherence challenges.

We first aimed to evaluate psychosocial differences between individuals indicated for MI and those not indicated for MI. Our quantitative analysis showed that participants in the MI Indicated group had higher perceived HIV vulnerability, greater depression symptom severity, and lower coping and self-efficacy compared to the MI Not Indicated group—offering insights for optimizing and personalizing PrEP adherence interventions. At week 12, we observed a difference in PrEP adherence that trended towards significance between the MI groups, suggesting lower adherence among participants needing MI compared to those who did not. We view this observation as noteworthy because it highlights early differences between the groups, suggesting that participants who required MI may have had slightly lower adherence, despite both groups showing overall high adherence. This difference, though not statistically significant, is valuable in the context of our analysis, which aimed to identify factors influencing the benefit of the tailored MI intervention.

Additionally, age and coping/self-efficacy were positively correlated with Week 12 PrEP adherence, while perceived vulnerability to HIV was negatively correlated, regardless of MI group. Texting behaviors further supported MI group classifications, with higher intervention engagement among the MI Not Indicated group. These results highlight the influence of psychosocial factors—such as stress, coping mechanisms, and depression—on both intervention engagement and adherence. Existing literature offers mixed findings: some studies identify depression and anxiety as barriers to PrEP adherence [40], while others find no association between depressive symptoms and daily oral PrEP adherence among gender minority adults [41, 42]. Aligning with our findings, another study cited concerns about PrEP efficacy and HIV protection as barriers for transgender women [43]. However, research focused specifically on TGNB individuals remains limited. Future interventions may benefit from enhanced psychosocial screening and the inclusion of coping-skills training or mental healthcare to better promote PrEP adherence in TGNB populations.

Our second aim was to assess whether constructs of the HBM were associated with PrEP adherence in a sample of TGNB adults. Specifically, demographic variables (age), psychological characteristics (coping and self-efficacy), and perceived susceptibility (perceived vulnerability to HIV) were significantly associated with PrEP adherence. These findings support the HBM as a useful explanatory model of PrEP adherence among TGNB individuals. Similar results were observed by Ashraf and Virk (2021) [44], who examined the HBM in antiretroviral medication adherence among people with HIV, finding that perceived HIV susceptibility, severity, and barriers were significant predictors of medication adherence, with 3% of their sample being transgender adults. Future studies could expand on these findings by including measures of perceived severity, as our secondary analysis did not assess this construct.

Our third aim was to qualitatively identify barriers and facilitators to PrEP adherence and intervention engagement among a subset of participants who completed MI. Common barriers included competing priorities, medical issues, text message burden, changes in routine, and emotional/social distress. Some participants discontinued PrEP due to perceived lowered risk of HIV or lack of access to medication. Similar barriers were identified in a meta-synthesis by Ching et al. 2020, which highlighted individual (e.g., HIV susceptibility, PrEP knowledge), organizational (e.g., cost, access, quality of services) and societal barriers (e.g., stigma, insurance, family/peer support) [45]. In contrast, Ching et al. (2020) found that perceived HIV risk was a facilitator of PrEP adherence. The barriers and facilitators reported in the MI call logs mostly reflect individual and interpersonal factors, possibly due to limitations of the MI prompts. However, societal and structural barriers to PrEP adherence—especially for TGNB folks—remain significant [12]. For Black and Latinx transgender women, stigma and medical mistrust are key barriers to PrEP uptake and adherence [46–48]. We hypothesize that the contradictory findings may stem from societal and structural barriers that were not measured in the current study.

Facilitators of PrEP adherence and intervention engagement in our study included finding iTAB text messages helpful as reminders, perceived psychological benefits from PrEP use, and utilization of additional adherence strategies (such as alarms and pillboxes). Establishing routines and integrating PrEP with other medications were also prominent facilitators. Complimentary to our results with a TGNB population, in a sample of gay and bisexual men, the use of multiple adherence strategies such as integrating PrEP into daily routines, using pillboxes, smartphone reminders, and partner support was associated with higher PrEP adherence [49]. Interestingly, while our sample found pairing PrEP with hormones to be a facilitator of PrEP adherence, other studies have suggested that TGNB folks may be fearful of PrEP/hormone interactions and prioritize hormone therapy over PrEP [50, 51], which is notable for both future providers and researchers to consider. This warrants the question, too, whether hormones are considered part of a routine, and if TGNB who take gender affirming hormones in pill form are more likely to pair their hormones with PrEP compared to TGNB taking hormone therapy by injection, gel, or patch. Future studies could also explore whether combining injectable PrEP with injectable hormones enhances PrEP adherence or reduces the need for daily text message reminders or MI.

Our quantitative findings suggest that perfect adherence to daily PrEP use is not strictly necessary for optimal protection against HIV, particularly in transgender and nonbinary individuals who may face unique challenges in maintaining daily medication routines. Biological TFV levels indicate that 4–5 doses per week can provide excellent adherence and protection. This aligns with the work of Anderson et al. (2012), which showed that missed doses (2 to 3 times per week) did not significantly reduce the effectiveness of daily PrEP among MSM [52]. The authors attributed this to the long intracellular half-life of tenofovir and its high concentrations in rectal tissue. Although the Anderson study did not specifically include transgender or nonbinary populations, these findings are relevant given that TGNB individuals face different barriers to adherence, such as stigma, access to care, and other gender-specific concerns. Our study further corroborates the evidence that flexible adherence strategies, such as intermittent dosing, may be an effective option for individuals who experience difficulties with daily dosing, thereby offering continued protection against HIV for those populations. These insights are critical for improving PrEP accessibility and adherence for TGNB individuals, who could benefit from more personalized approaches to HIV prevention.

### Integrating Qualitative and Qualitative Results

Qualitative insights from MI call logs complemented our quantitative findings, providing a richer understanding of the barriers and facilitators influencing PrEP adherence among TGNB individuals. Our qualitative data highlight challenges related to changes in routine, which dovetailed with our quantitative results showing difficulties in coping and self-efficacy. Together, this suggests that individuals with lower coping and self-efficacy are less likely to effectively respond to changes in their routines, leading to higher risk for non-adherence and lowered engagement with the intervention. Although the relationship between routines, coping and self-efficacy, and PrEP adherence has not been examined specifically among TGNB, Owens et al. 2020 [53] observed that in a sample of MSM, participants who had high self-efficacy and high self-reliance to existing daily routines were subsequently more adherent to PrEP. Moving forward, future research should explore interventions tailored to address routine disruptions, enhance coping strategies, and bolster self-efficacy among TGNB individuals to improve PrEP adherence and overall health outcomes.

The emotional and social distress documented in the MI call logs corroborated the elevated levels of stress and depression identified among the MI Indicated group in our quantitative analysis. This underscores the importance of addressing mental health concerns within PrEP adherence interventions, particularly among TGNB who may be facing multiple stressors and challenges in their daily lives [54]. Future research endeavors should focus on developing and implementing interventions that integrate mental health support components to enhance PrEP adherence among TGNB individuals, thereby addressing the complex interplay between mental health and medication adherence in this population.

Our study found a surprising negative association between perceived vulnerability to HIV and PrEP adherence such that higher perceived vulnerability was associated with lower PrEP adherence. Typically, perceived vulnerability to HIV and PrEP adherence are positively associated [55–57]. However, perceptions of risk and vulnerability are highly complex, and change over time depending on behaviors, which may not be captured by a scaled questionnaire [58, 59]. We hypothesize that the observed inverse relationship may be linked to the heightened stress and depression levels among the MI indicted group. Despite recognizing the increased HIV risks and prevalence within the TGNB community, individuals may have been overwhelmed by systemic barriers and daily challenges, limiting their capacity to effectively address those risks. Future investigations might explore further the nuanced interplay between perceived vulnerability, mental health factors, and systemic barriers within the TGNB community.

### Limitations

The findings of this study should be interpreted in light of several limitations. Firstly, the relatively small sample size (N = 128) was restricted to the active intervention group exclusively. For a more comprehensive comparative investigation between the active and control groups, we refer readers to Morris et al., 2022 [30]. Additionally, high attrition rates presented statistical challenges for the week 48 analysis. However, it is noteworthy that retention rates at both Week 12 (N = 88) and Week 48 (N = 42) showed no significant differences between MI Engagement groups. Nonetheless, we acknowledge the potential impact of attrition on our analysis. Also noteworthy is that our qualitative analysis was confined to MI call logs, which would have been more robust had the MI sessions been audio recorded. Audio recordings would have facilitated a more in-depth qualitative analysis. In assessing the efficacy of the HBM framework, it is important to note that our study lacked one of the constructs, perceived severity. As a secondary analysis, we could not incorporate a measure into the completed intervention, but future studies should incorporate a perceived severity measure. Future interventions may benefit from a priori selecting measures to capture all constructs of the HBM framework. Another important limitation is that both “no” responses and non-responses to adherence text messages were coded as missed doses, which may not fully capture the reasons for non-response. Non-responses could reflect factors unrelated to adherence, such as participant burden or disengagement, and may differ meaningfully from explicit reports of non-adherence. MI telephone sessions also represented an opportunity for participants to indicate whether their non-responsive was due to message fatigue/something else vs. non-adherence to PrEP. Regarding the generalizability of our findings, our sample primarily consisted of transgender women, with nonbinary individuals comprising the smallest group. This imbalance limits the generalizability of our findings to the TGNB community broadly but nonetheless constitutes an important contribution to the emerging literature on this underserved population. Our study sought to explore potential differences in PrEP adherence between transgender and non-binary individuals; however, a sensitivity analysis revealed that our sample was not adequately powered to detect meaningful differences between these subgroups. As a result, we cannot confidently draw conclusions regarding adherence variations across the TGNB populations. This limitation underscores the need for larger, more focused studies to better understand adherence patterns among TGNB individuals. Future research should consider examining these subgroups separately to inform more tailored interventions and improve PrEP adherence for these diverse populations. Additionally, we acknowledge the inclusion of the MI Indicated group variable in the final model despite its lack of a significant relationship with PrEP Adherence. This decision was deliberate, serving as our a priori predictor to assess the effect of MI engagement on PrEP adherence. Finally, as a cross-sectional study, we were unable to establish causal relationships between our variables.

### Implications

Our study emphasizes the need for personalized interventions for TGNB individuals, covering PrEP adherence, mental health, psychological traits, and HIV susceptibility perception. Future interventions could use the HBM as a guide for assessing individual-level factors that potentially influence PrEP adherence. Retaining participants in such interventions is challenging, necessitating innovative strategies focused on long-term engagement, including addressing TGNB-specific mental health needs and stressors. Both researchers and policymakers must prioritize the inclusion of TGNB individuals in PrEP access and implementation efforts. To advance the goals outlined by the Ending the HIV Epidemic initiative, it is imperative to enhance access to facilitators and address barriers that hinder uptake and adherence within this population, as identified in our project.

## Conclusions

Overall, the synthesis of quantitative and qualitative data in this study highlights the complex web of factors influencing PrEP adherence and intervention engagement among TGNB individuals. Our results underscore the necessity of developing comprehensive, culturally sensitive PrEP interventions tailored to TGNB communities. These interventions should not only address mental health concerns but also encompass psychological characteristics and perceived susceptibility to HIV. Moreover, integrating strategies to promote lifestyle stability and consistency could bolster intervention engagement, thereby maximizing its effectiveness and long-term benefits of PrEP adherence interventions.
